# A scoring model based on MRI features for predicting early recurrence after surgical resection of hepatocellular carcinoma

**DOI:** 10.3389/fsurg.2025.1488276

**Published:** 2025-08-01

**Authors:** Yi-Jing Wang, Jian-Xia Xu, Tian-Yu Ke, Bao-Na Li, Xiao-Zhong Zheng, Jun-Yi Xiang, Shu-Feng Fan, Xiao-Shan Huang

**Affiliations:** ^1^ Department of Radiology, HangZhou Red Cross Hospital, Hangzhou, China; ^2^ Department of Radiology, The Second Affiliated Hospital of Zhejiang Chinese Medical University, Hangzhou, China; ^3^ Department of Radiology, Qinghai Provincial People’s Hospital, Xining, Qinghai, China

**Keywords:** hepatocellular carcinoma, early recurrence, MRI, surgical resection, scoring model

## Abstract

**Objectives:**

Based on MRI features, a scoring model was constructed to predict early recurrence after surgical resection of hepatocellular carcinoma (HCC).

**Methods:**

A total of 310 patients from two centers with HCC (212 in the training cohort, 98 in the validation cohort) were collected from January 2017 to October 2023, all patients underwent preoperative MRI-enhanced examinations and were pathologically diagnosed after resection and were divided into early recurrence group and non-early recurrence group based on follow-up results. Clinical, laboratory, and MRI features of patients were collected and subjected to statistical analysis. Univariate analysis and multivariable analysis were used to identify independent predictive factors. The independent predictive factors for early recurrence of liver cancer were weighted using regression coefficient-based scores and construct a score model integrating preoperative variables. Subsequently, receiver operating characteristic (ROC) curves and calibration curves were created to evaluate the performance of the scoring model. The overall score distribution was divided into four groups to show the probability of distinguishing early recurrence.

**Results:**

After multifactor analysis, tumor number, tumor margin, peritumoral enhancement, and macrovascular invasion were identified as independent predictors of early recurrence in preoperative variables. Among them, the tumor margin predictor was assigned 3 points, while the remaining predictors were each assigned 2 points. With a cutoff value of 3.5 points, the ROC value of the score model were 0.873 and 0.847, with sensitivities of 83.9% and 81.3%, and specificities of 77.8% and 73.8%. According to the scores, the predictive ability of early recurrence increased across the four groups.

**Conclusions:**

The established scoring model effectively predicts early recurrence after surgical resection of HCC. The simplicity of the scoring model facilitates clinical application, aiding in the development of personalized treatment plans before surgery.

## 1 Introduction

Hepatocellular carcinoma (HCC) is one of the most prevalent malignant tumors globally, ranking sixth in incidence and third in cancer-related mortality. Furthermore, both the incidence and mortality of HCC are increasing annually (1, 2). For HCC patients, liver resection and liver transplantation remain the primary curative treatments (3, 4). While liver transplantation offers the definitive advantage of removing both the tumor and the diseased liver, the demand for donor organs greatly exceeds the available supply. As a result, liver resection is widely regarded as the first-line treatment option for HCC patients with preserved liver function, whereas liver transplantation is recommended for those with decompensated cirrhosis (5). Unfortunately, due to the aggressive nature and immunosuppressive microenvironment of HCC (6), the 5-year recurrence rate following surgical resection can be as high as 70%. Notably, patients who experience early recurrence, defined as recurrence within the first two years post-surgery, face a particularly poor prognosis (7). Therefore, accurately predicting early recurrence is critical for guiding preoperative treatment decisions in HCC patients.

To assess the prognosis of HCC patients, researchers have developed numerous predictive models, with most studies focusing on preoperatively predicting early recurrence after hepatectomy for liver cancer. These models incorporate preoperative clinical and imaging indicators for recurrence prediction, such as predictive models based on imaging features or radiomics, as well as models based on clinical-imaging features, and so on (8–10). Preoperative prediction aids in formulating clinical treatment plans. Currently, omics is a hot research topic, but due to its specificity and complexity, many research findings cannot be effectively applied in clinical practice. Moreover, due to variations in the predictive capabilities of different models and indicators, there is still a lack of a simple and effective method to assess postoperative recurrence of liver cancer.

Our team has developed a scoring model and published a series of articles utilizing it (11–15). These articles demonstrate that the scoring model is effective in disease discrimination and prognosis prediction, proving it to be reliable and user-friendly. Therefore, this study aims to construct a preoperative scoring model for predicting early recurrence after liver cancer surgery based on imaging features.

## 2 Materials and methods

### 2.1 Patient population

**Figure 1:**
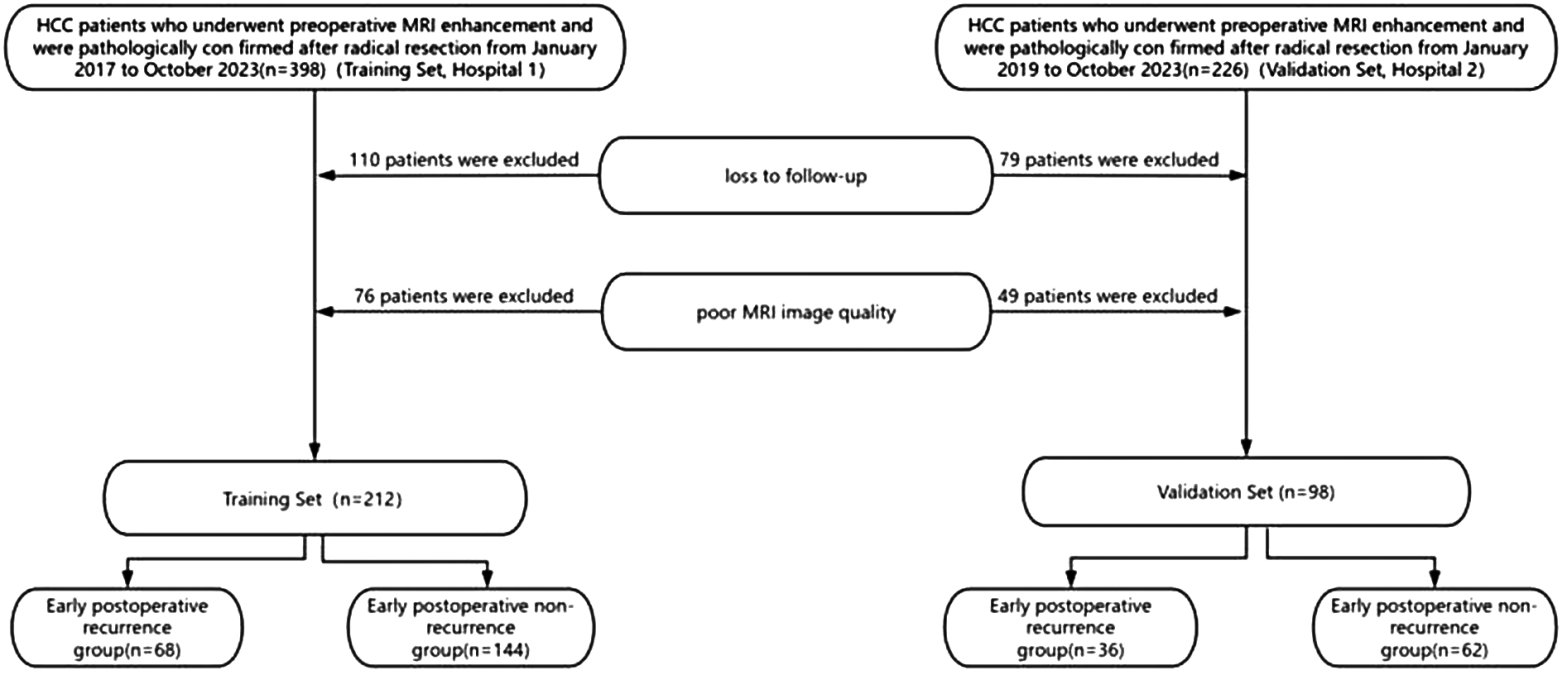
Flow diagram of participants.

We retrospectively collected data from hepatocellular carcinoma (HCC) patients at two centers from January 2017 to October 2023. Patients from the Second Affiliated Hospital of Zhejiang Chinese Medical (hospital 1), were assigned as the training cohort. Patients from the Qinghai Provincial People's Hospital (hospital 2) were assigned as the validation cohort. Inclusion criteria comprised patients who underwent abdominal enhanced MRI examination within 2 weeks before surgery, received curative resection for HCC, pathologically confirmed as HCC, and had a follow-up period of more than 2 years after surgery or experienced recurrence within less than 2 years of follow-up. Exclusion criteria included patients who underwent interventions such as ablation, chemotherapy, targeted therapy, immunotherapy, or other treatments before surgery, as well as those who died perioperatively. A total of 624 HCC patients were initially included, but due to loss to follow-up, poor imaging quality etc., only 310 patients were ultimately included. A total of 212 patients in the training cohort—68 patients in the early recurrence group and 144 patients in the non-recurrence group. A total of 98 patients in the validation cohort—36 patients in the early recurrence group and 62 patients in the non-recurrence group (Figure 1). The study protocol was approved by the Ethics Committee of the Second Affiliated Hospital of Zhejiang Chinese Medical University and the Qinghai Provincial People's Hospital.

### 2.2 Follow-up surveillance and clinical endpoint

All patients were followed up for at least 2 years after curative resection. In the first month postoperatively, tumor recurrence was screened using serum AFP level assessment, enhanced chest and abdominal CT, or abdominal MRI. Subsequently, follow-up visits were scheduled every 3 months in the first year and every 6 months thereafter. The review endpoint date was October 10, 2023.

The study endpoint was early recurrence, defined as the occurrence of one or more of the following events within 2 years after curative resection surgery: (a) presence of new liver lesions with typical HCC radiological features; (b) histologically confirmed HCC on biopsy or postoperative pathology or non-typical imaging findings suggestive of tumor staining after transarterial chemoembolization (TACE); (c) extrahepatic metastasis confirmed by typical radiological features or histological analysis.

### 2.3 Clinical data

Query and record relevant clinical information of patients with liver cancer before surgery from our hospital's electronic medical record system, including patient gender, age, presence of liver cirrhosis, AFP (alpha-fetoprotein) level, ALT (alanine aminotransferase) level, AST (aspartate aminotransferase) level, ALP (alkaline phosphatase) level, TBIL (total bilirubin) level, and ALB (albumin) level.

### 2.4 MRI imaging equipment and parameters

The MRI scanners used in this study were the Siemens Magnetom Avanto 1.5 T (hospital 1) and the Siemens Skyra 3.0 T (hospital 2). All patients were required to fast for at least 4 h before the examination and undergo breath-holding training prior to scanning. The scanning range extended from the top of the diaphragm to the lower edge of the liver. The scanning parameters (hospital 1) were set as follows: (1) T1WI: TR = 5.6 ms, TE = 2.1 ms, slice thickness = 3.5 mm, interslice gap = 5 mm; (2) T2WI: TR = 3,100 ms, TE = 76 ms, slice thickness = 4.5 mm, interslice gap = 5 mm; (3) DWI: TR = 6,900 ms, TE = 59 ms, slice thickness = 4.5 mm, interslice gap = 5 mm, with b-values of 50 and 1000 s/mm^2; (4) DCE-MRI TR = 3.8 ms, TE = 1.5 ms, slice thickness = 3 mm, interslice gap = 0 mm; And the scanning parameters (hospital 2) were set as follows: (1) T1WI: TR = 3.5 ms, TE = 1.5 ms, slice thickness = 5 mm, interslice gap = 1 mm; (2) T2WI: TR = 2,800 ms, TE = 100 ms, slice thickness = 5 mm, interslice gap = 1 mm; (3) DWI: TR = 8,500 ms, TE = 80 ms, slice thickness = 5 mm, interslice gap = 1 mm, with b-values of 400 and 1000 s/mm^2; (4) DCE-MRI TR = 4.5 ms, TE = 2 ms, slice thickness = 3 mm, interslice gap = 0.5 mm. Liver multiphase dynamic contrast-enhanced imaging: Gd-DTPA (gadolinium-diethylenetriamine pentaacetic acid) was used as the contrast agent in all cases. It was administered intravenously at a dose of 0.1 mmol/kg and an injection rate of 2 ml/s, followed by a 20 ml flush of normal saline. Contrast-enhanced scans were acquired during the arterial phase, portal venous phase, and delayed phase.

### 2.5 Qualitative analysis of MR images

Qualitative analysis of MR image features was independently conducted by two abdominal radiologists with 10 and 20 years of diagnostic experience in abdominal imaging, respectively. The radiologists were blinded to radiological and pathological reports. Both physicians observed the features twice, with the second observation occurring 2 weeks after the first. On a per-patient basis, the reviewers evaluated 10 imaging features that have been reported to describe HCC (16–18), including: Tumor number; Tumor size, defined as the maximum diameter on axial delayed-phase images; Arterial phase rapid enhancement (Nonrim-like enhancement of the tumor in the arterial phase unequivocally greater in whole or in part than the liver); portal phase rapid clearance (Nonperipheral visually assessed temporal reduction in the enhancement of the tumor in whole or in part relative to composite liver tissue in the portal venous phase or delayed phase); Peritumoral enhancement (refers to the wedge-shaped or irregular enhancement around the tumor in the late arterial phase or early portal venous phase); Intratumoral hemorrhage (Intralesional or perilesional hemorrhage in the absence of biopsy, trauma, or intervention); Intratumoral necrosis (Presence of nonenhancing area in a solid mass, not attributable to a cystic component, prior treatment, or intralesional hemorrhage); Tumor margin (whether the tumor margin is smooth and uniformly enhanced in the portal phase or delayed phase); Pseudo capsule (A fibrous reactive band surrounding the tumor, visible across multiple imaging phases, that resembles a “capsule” in both morphology and enhancement characteristics, yet is not a true anatomical capsule); macrovascular invasion (unequivocally enhancing soft tissue in vein).

### 2.6 Statistical analysis

Quantitative data was presented as the mean ± standard deviation (M ± SD) when the data distribution was normal, or median and interquartile range (IQR) suitable for the data of abnormal distributions, and enumeration data was recorded as frequency (percentages), as appropriate. The chi-square or Fisher's exact test was used for categorical variables and the Student t or Mann–Whitney *U*-test for continuous variables in univariate analysis. A value of P relaxed to less than 0.05 was considered to indicate a significant difference in univariate analysis. Include variables with *P* < 0.05 from univariate analysis in multivariate analysis. Any variable with a *P* value ≤ 0.05 was retained in the final model. To derive a simple-to-compute and optimal score, we converted regression coefficients to weighted scores by dividing each regression coefficient by one-half of the smallest coefficient and rounding to the nearest integer or taking the integer part. For each patient, the individual score corresponding to the predictors was summed together to produce an overall score. ROC curve and calibration curve are used to evaluate the performance of the model. *P* value of < 0.05 was considered statistically significant. All statistical analysis were performed using SPSS software (IBM SPSS Statistics Version 25.0; Origin 2019b).

## 3 Result

### 3.1 Demographic characteristics of the patients

There was no significant difference in clinical characteristics between the training cohort and the validation cohort (Table 1). In the training cohort, no statistically significant differences were observed in demographic characteristics and laboratory parameters between the early recurrence group and the non-recurrence group after liver cancer resection (*P* > 0.05, Table 2).

**Table 1:** Comparison of the base line characteristics for HCC patients after resection between the training and validation cohorts.

Characteristics	Training cohort	Validation cohort	*p*-value
	(*n* = 212)	(*n* = 98)	
Age	55.56 ± 10.97	54.12 ± 9.26	0.212
Gender			0.187
Male	167 (79%)	72 (73%)	
Female	45 (21%)	28 (27%)	
Liver cirrhosis			0.312
Absent	146 (69%)	73 (74%)	
Present	66 (31%)	25 (26%)	
Child-Pugh grade			
A	161 (76%)	69 (70%)	0.378
B	51 (24%)	29 (30%)	
AFP (ng/ml)			0.315
>100	75 (35%)	29 (30%)	
≤100	137 (65%)	69 (70%)	
ALT(U/L)	61.62 ± 82.74	63.11 ± 78.09	0.832
AST(U/L)	66.77 ± 97.79	62.41 ± 93.53	0.631
ALP(U/L)	99.04 ± 121.61	101.77 ± 118.82	0.811
TBIL (umol/L)	22.22 ± 31.95	21.41 ± 18.38	0.741
ALB(g/L)	42.60 ± 19.68	40.15 ± 20.33	0.259
Tumor diameter			0.341
≥5cm	63 (30%)	24 (24%)	
<5cm	149 (70%)	74 (76%)	
Tumor number			0.224
Solitary	35 (17%)	11 (11%)	
Multiple	177 (83%)	87 (89%)	
Tumor margin			0.851
Nonsmooth	82 (39%)	39 (40%)	
Smooth	130 (61%)	59 (60%)	
Pseudo capsule			0.535
Absent	68 (32%)	28 (29%)	
Present	144 (68%)	70 (71%)	
Intratumoral hemorrhage			0.409
Absent	43 (20%)	16 (16%)	
Present	169 (80%)	82 (84%)	
Intratumoral necrosis			0.242
Absent	106 (50%)	42 (43%)	
Present	106 (50%)	56 (57%)	
Arterial rapid enhancement			0.580
Absent	101 (48%)	50 (51%)	
Present	111 (52%)	48 (49%)	
Portal venous rapid clearance			0.348
Absent	133 (63%)	56 (57%)	
Present	79 (37%)	42 (43%)	
Peritumoral enhancement			0.572
Absent	109 (51%)	47 (48%)	
Present	103 (49%)	51 (52%)	
Macrovascular invasion			0.197
Absent	184 (87%)	90 (92%)	
Present	28 (13%)	8 (8%)	

**Table 2:** The clinical and imaging data between the early recurrence group and the non-recurrence group in the training cohort.

Characteristics	Recurrence group (*n* = 68)	Non-recurrence group (*n* = 144)	Univariate analysis
			*p* value
Age	53.69 ± 9.76	56.35 ± 11.50	0.247
Gender			0.884
Male	53 (78%)	114 (79%)	
Female	15 (22%)	30 (21%)	
Liver cirrhosis			0.239
Absent	41 (61%)	105 (73%)	
Present	27 (39%)	39 (27%)	
Child-Pugh grade			
A	53 (78%)	108 (75%)	0.422
B	15 (22%)	36 (25%)	
AFP (ng/ml)			0.169
>100	30 (44%)	45 (31%)	
≤100	38 (56%)	99 (69%)	
ALT(U/L)	52.19 ± 69.74	66.12 ± 88.18	0.423
AST(U/L)	50.41 ± 69.59	74.47 ± 108.43	0.235
ALP(U/L)	96.36 ± 79.82	100.37 ± 136.82	0.873
TBIL (umol/L)	28.15 ± 54.04	19.43 ± 11.39	0.222
ALB(g/L)	43.53 ± 11.67	42.15 ± 21.96	0.727
Tumor diameter			0.368
≥5cm	24 (36%)	39 (27%)	
<5cm	44 (64%)	105 (73%)	
Tumor number			0.001
Solitary	23 (34%)	12 (8%)	
Multiple	45 (66%)	132 (92%)	
Tumor margin			<0.001
Nonsmooth	47 (69%)	35 (24%)	
Smooth	21 (31%)	109 (76%)	
Pseudo capsule			0.127
Absent	15 (22%)	53 (37%)	
Present	53 (78%)	91 (63%)	
Intratumoral hemorrhage			0.390
Absent	17 (25%)	26 (18%)	
Present	51 (75%)	118 (82%)	
Intratumoral necrosis			0.406
Absent	30 (44%)	76 (53%)	
Present	38 (56%)	68 (47%)	
Arterial rapid enhancement			0.481
Absent	32 (47%)	69 (48%)	
Present	36 (53%)	75 (52%)	
Portal venous rapid clearance			0.057
Absent	34 (50%)	99 (69%)	
Present	34 (50%)	45 (31%)	
Peritumoral enhancement			0.004
Absent	49 (72%)	60 (42%)	
Present	19 (28%)	84 (58%)	
Macrovascular invasion			<0.001
Absent	47 (69%)	137 (95%)	
Present	21 (31%)	7 (5%)	

### 3.2 MRI imaging features

MRI imaging features that showed statistically significant differences in univariate analysis, including Tumor number, Tumor margin, Peritumoral enhancement, and macrovascular invasion (Table 2). Further multivariable logistic regression revealed that Tumor number, Tumor margin, Peritumoral enhancement, and macrovascular invasion are independent predictors for early postoperative recurrence after HCC resection (Table 3).

**Table 3:** Multivariate regression analysis for predicting early recurrence after HCC resection and the weighted score of independent predictors.

Characteristics	B	P	OR	95% CI	Weighted Score
Tumor number	1.363	0.048	0.256	0.066–0.990	2
Tumor margin	1.908	0.001	0.148	0.049–0.447	3
Peritumoral enhancement	1.397	0.015	0.247	0.081–0.760	2
Macrovascular invasion	1.591	0.015	0.204	0.058–0.717	2

### 3.3 Development of the predictive model

**Figure 2:**
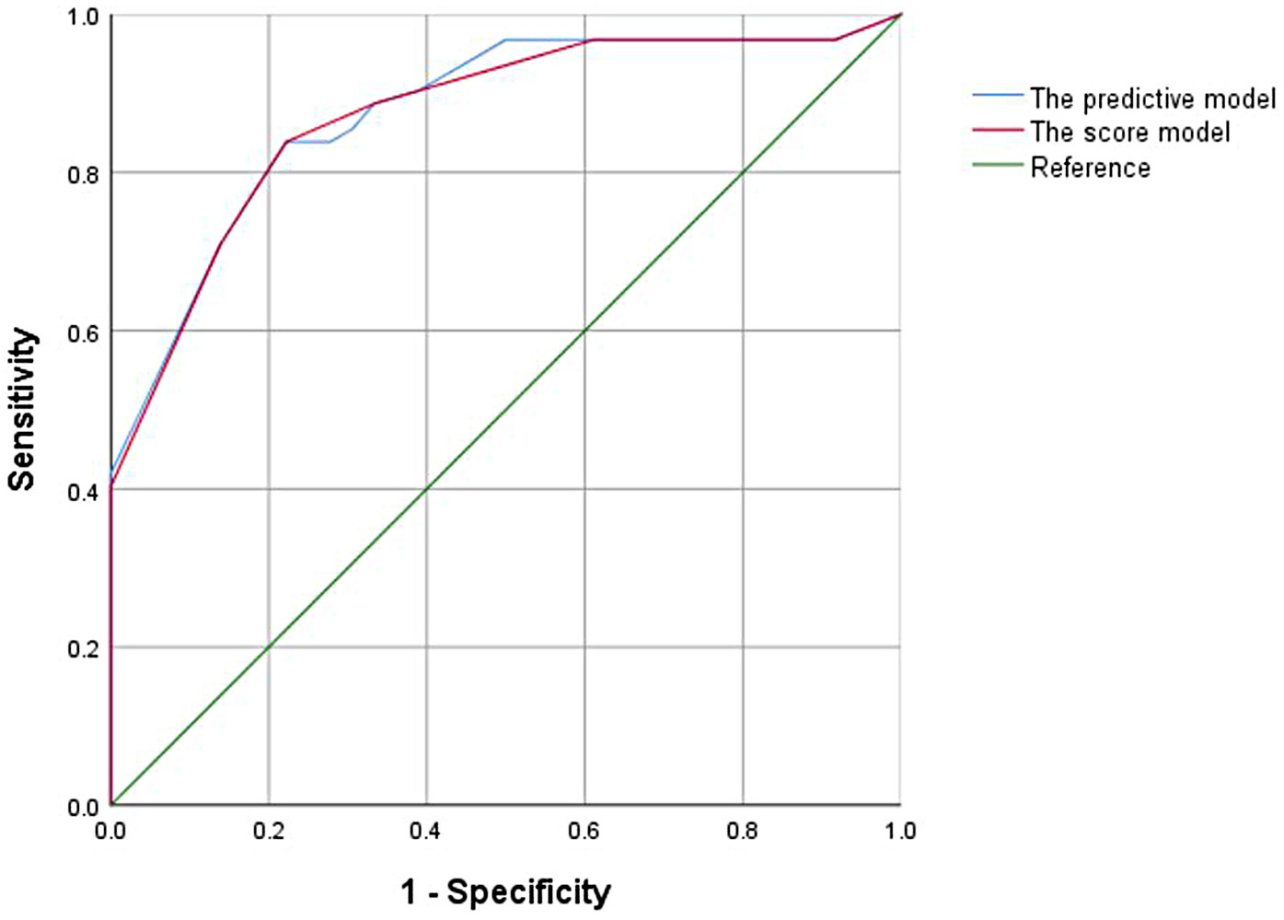
ROC curves for the preoperative scoring model.

In the univariate analysis of the training cohort, variables with *P* < 0.1 were selected for collinearity diagnostics, which demonstrated no significant multicollinearity (VIF < 5). Subsequently, through multivariate logistic regression analysis, tumor number, tumor margin, peritumoral enhancement, and macrovascular invasion showed statistically significant differences (*P* < 0.05). A traditional predictive model was constructed using these four features for preoperative prediction. The Hosmer–Lemeshow goodness-of-fit test showed good calibration of this predictive model (*p* = 0.427), and the model exhibited good performance with an AUC of 0.876 (95% CI, 0.817–0.923) (Figure 2).

### 3.4 Development of the scoring system

**Figure 3:**
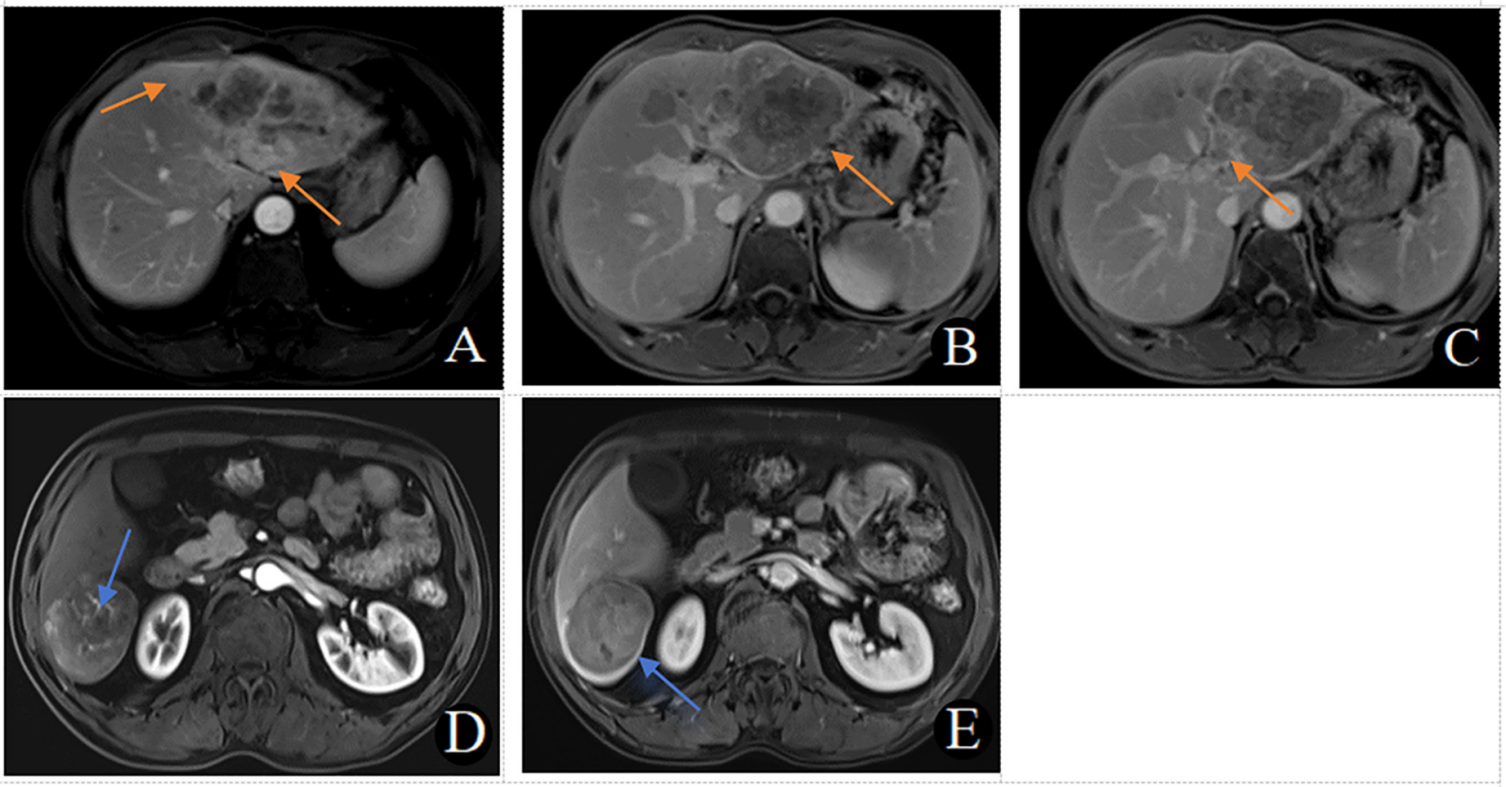
**(A–C)** MRI in a 56-year-old male patient with early recurrence (within 2 years) after liver cancer resection. **(A)** Arterial phase late peritumoral enhancement is seen (orange arrow). **(B)** Two intrahepatic lesions are present, with the larger lesion showing irregular tumor margins in the portal venous phase (orange arrow). **(C)** A filling defect in the left branch of the portal vein and the formation of a cancerous thrombus is observed in the portal venous phase (orange arrow). This patient scored 9 points. **(D, E)** MRI in a 54-year-old male patient without early recurrence (within 2 years) after liver cancer resection. D. No peritumoral enhancement is observed in the arterial phase, with visible intratumoral arteries. **(E)** The tumor margins are smooth, with uniform enhancement in the portal venous phase. This patient scored 0 points.

**Figure 4:**
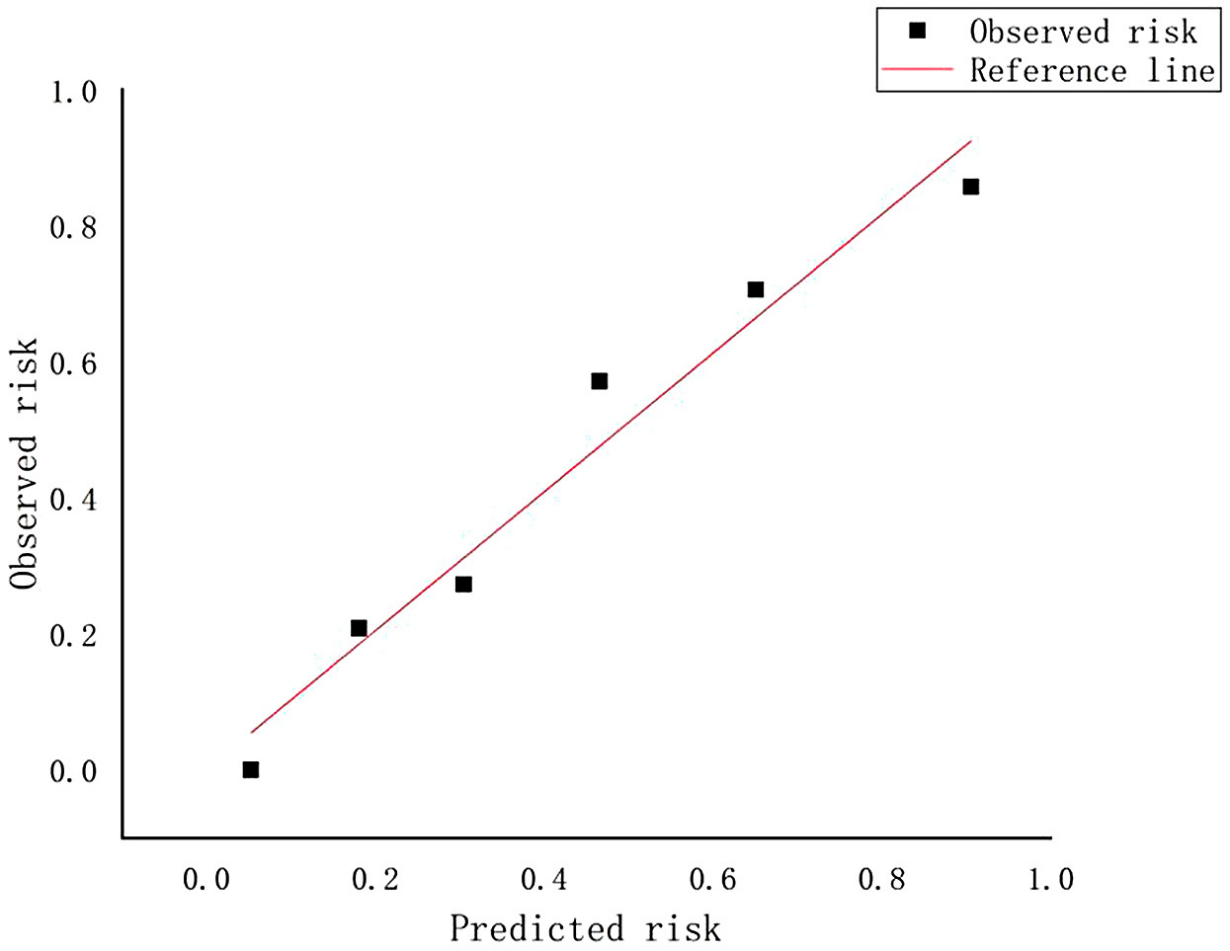
Calibration curve for the scoring model.

A weighted score was assigned to each independent predictor associated with early recurrence after HCC resection as follows: The preoperative model: 2 points, tumor number; 3 points, tumor margin; 2 points, peritumoral enhancement; 2 points, macrovascular invasion (Table 3). For each patient, the individual scores that correspond to all the independent predictors were summed together to produce an overall score, which was named as score model. In our study, the score range for the preoperative model is 0–9 points, with a median score of 2 points. The higher the score, the greater the probability of early recurrence after liver cancer resection (Figure 3). The Hosmer-Lemeshow goodness-of-fit test indicated that the scoring model had good calibration (*p* = 0.790). Additionally, we plotted the calibration curve for the scoring model, which demonstrated good concordance (Figure 4). The AUC of this discriminative scoring system, as measured by the receiver operating characteristic (ROC) curve analysis, was 0.873 (95% CI, 0.803–0.943) (Figure 2). Using 3.5 as the cutoff value, the model showed high predictive efficiency, with a sensitivity of 83.9%, specificity of 77.8%, and accuracy of 80.0% (Table 4). A comparison of the ROC curves showed no significant difference between the main predictive model and the scoring model (*p* = 0.132), indicating that the scoring model effectively utilized the predictive model's values and could accurately predict early recurrence after liver cancer resection.

**Table 4:** The performance of the scoring system in the training and validation cohorts.

Metrics	Training cohort	Validation cohort
Sensitivity	0.839	0.813
Specificity	0.778	0.738
Positive likelihood ratio	3.78	3.12
Negative likelihood ratio	0.21	0.26
PPV	0.712	0.703
NPV	0.930	0.890
ACC	0.800	0.772

### 3.5 Validation of the established scoring system

To validate the predictive performance of the scoring model, we conducted external validation, and the results of the scoring system were satisfactory. The Hosmer-Lemeshow goodness-of-fit test demonstrated good calibration (*p* = 0.694). In the validation cohort, the AUC of the scoring system was 0.847 (95% CI, 0.791–0.921). At a cutoff value of 3.5, the model's sensitivity, specificity, and accuracy were 81.3%, 73.8%, and 77.2%, respectively (Table 4), similar to the results in the training cohort.

To apply the score system conveniently in clinical work, we further divided scores into 4 separate groups: preoperative score: ≥0 point and ≤2 points, >2 points and ≤4 points, >4 points and ≤6 points and >6 points and ≤9 points. The predictive probability of early recurrence after HCC resection increases as stage increase of the score (Table 5).

**Table 5:** Predictive probability of early recurrence in different score ranges in the training and validation cohorts.

Score range	Training cohort			Validation cohort		
	Number of early recurrence	Total number	Predictive probability of early recurrence	Number of early recurrence	Total number	Predictive probability of early recurrence
≥0 and ≤2	1	110	1%	5	49	10%
>2 and ≤4	15	38	40%	7	18	39%
>4 and ≤6	27	36	75%	12	17	71%
>6 and ≤9	25	28	89%	12	14	86%

## 4 Discussion

The present study aimed to develop a scoring model for predicting early recurrence after surgical resection of hepatocellular carcinoma (HCC), based on MRI features. The results indicate that the model with AUC values of 0.873 effectively predict early recurrence, demonstrating a good predictive capability.

Previous studies have repeatedly emphasized the crucial role of imaging features in prognosis evaluation for liver cancer patients. This study further identified several key factors significantly associated with early HCC recurrence, including the number of tumors, tumor margin, peritumoral enhancement, and macrovascular invasion. These findings are consistent with earlier reports (19). In particular, peritumoral enhancement is often related to compensatory arterial blood flow, and the enhanced area may represent the initial site of micrometastases (20). Moreover, an irregular tumor margin—often manifested as small protrusions at the periphery—may indicate a multi-nodular, fused growth pattern at the histological level (21). Outward tumor growth leading to blurred margins is considered a key imaging sign of portal vein invasion and intrahepatic spread. Thus, irregular tumor margins driven by tumor heterogeneity and anisotropic growth commonly imply infiltration into the surrounding liver parenchyma, correlating closely with higher malignancy and a tendency toward early recurrence (22).

A study by Lee et al. (23) reported that combining peritumoral enhancement and irregular tumor margins yielded a specificity of 92.5% (124/134) in predicting microvascular invasion (MVI). Patients exhibiting these two imaging features showed a significantly increased rate of early recurrence. Additionally, Xu et al. (24) demonstrated that HCC patients with these imaging findings had shortened progression-free and overall survival. Huang et al. (25) found that peritumoral enhancement and irregular tumor boundaries serve as indicators of poor tumor differentiation in HCC, which is closely associated with early tumor recurrence and worse survival outcomes (26). In line with these findings, our study also noted that patients with both peritumoral enhancement and irregular tumor margins experienced higher early recurrence rates.

The impact of macrovascular invasion on postoperative HCC recurrence is well-established. Chen et al. (27) identified macrovascular invasion as an independent predictor of early recurrence following hepatic resection, and our findings are consistent with their conclusions. In addition, some literature (9, 16) suggests that a higher number of tumors may reflect greater tumor aggressiveness and activity, thus increasing the risk of early recurrence. Wang et al. (28) also showed that tumor number was closely associated with poor prognosis in HCC patients. Our study similarly found that patients with two or more tumor nodules were more prone to early recurrence, consistent with most existing studies. However, it is worth noting that some reports suggest that tumor number may not be a key predictor of early recurrence, but rather the only independent predictor of late recurrence (16).

Given the high rate of early recurrence following surgical resection of HCC, many researchers have devoted efforts to identifying its risk factors. Owing to the heterogeneity of HCC, patient outcomes vary significantly. Although genetic characteristics can improve prognostic assessment, they have not yet been integrated into routine clinical practice (3). Conversely, the power and potential of imaging data have increasingly gained recognition in oncology (29). Traditional imaging assessments have mostly been descriptive and cannot quantitatively predict risk. While radiomics—an emerging field—holds promise, the complexity of current methods poses challenges to their widespread clinical application.

In this context, our scoring model applies weighted scoring to all independent predictive factors. This approach not only reflects the relative importance of each predictor but also allows for the direct calculation of an individual patient's prognostic score, thereby quantifying the predicted risk of early recurrence. Our results show that patients with a score >4 have a 75% and 71% probability of early recurrence in the training and validation cohorts, respectively, and are classified as a high-risk group; patients with a score >2 and ≤4 have a 40% and 39% probability of early recurrence in the training and validation cohorts, respectively, and are classified as a medium-risk group; patients with a score ≥0 and ≤2 have a 1% and 10% probability of early recurrence in the training and validation cohorts, respectively, and are classified as a low-risk group. According to the Chinese Clinical Practice Guideline for Primary Liver Cancer(2024 Edition) (30), we recommend that patients undergo their first postoperative imaging follow-up within 3 months, followed by a follow-up every 3 months. After 2 years, the interval can be extended to 3–6 months. For high-risk patients, the frequency of follow-up may be increased, while low-risk patients may reduce the frequency of follow-up.

Compared to other models, our scoring system not only provides quantitative assessment and simplicity in operation, but also achieves an efficacy (AUC = 0.873) that is comparable to or even better than existing models (AUC = 0.7316^19^, 0.7710^10^, 0.9499^9^). This scoring model assists clinicians in making preoperative decisions and guiding postoperative management. Its straightforward nature enhances clinical applicability and facilitates seamless integration into daily practice. In both transplant and non-transplant settings, recurrence risk stratification plays a critical role in guiding treatment decisions. A recent study by Mazzotta et al. (31) highlighted that even among patients eligible for liver transplantation under the AFP score ≤2 criteria, those with ≥5 nodules during the waiting period had significantly worse outcomes, suggesting the need for dynamic reassessment of recurrence risk before transplant. These findings underscore the importance of accurate, preoperative recurrence prediction tools—like our imaging-based scoring model—in identifying high-risk patients earlier and more accurately. This, in turn, may help clinicians tailor postoperative strategies, guide transplant candidacy, or intensify surveillance, thereby demonstrating potential value across both surgical resection and transplant pathways.

Despite these encouraging results, several limitations must be acknowledged. First, this retrospective design may introduce selection bias, and future prospective, multicenter studies are warranted. Second, this study did not examine the impact of surgical factors on patient prognosis. Previous research has shown that intraoperative blood loss, the extent of hepatic resection, and anatomical vs. non-anatomical resection can all influence patient outcomes. Third, the follow-up period in our study was relatively short, and late recurrence was not evaluated. Extending the follow-up duration in future studies would allow a more comprehensive assessment of imaging risk factors in predicting HCC recurrence. Fourth, most patients in this study were HBV-infected. It is well known that, while most patients with combined hepatocellular-cholangiocarcinoma in Eastern countries are affected by HBV, its incidence is much lower in Western populations; hence, the generalizability of our conclusions may be limited. Finally, as imaging technology rapidly advances, new techniques capable of capturing more nuanced tumor information continue to emerge. Therefore, our scoring model requires ongoing optimization and validation over time.

In conclusion, this study developed a scoring model for predicting early recurrence after surgical resection of HCC. The model demonstrated effectiveness, strong predictive ability. Due to the simplicity and efficacy of the scoring model, it holds promise for clinical application, aiding clinicians in formulating personalized treatment plans.

## Data Availability

The original contributions presented in the study are included in the article/Supplementary Material, further inquiries can be directed to the corresponding author.
